# A Vehicle-Assisted Computation Offloading Algorithm Based on Proximal Policy Optimization in Vehicle Edge Networks

**DOI:** 10.1007/s11036-022-02029-y

**Published:** 2022-09-12

**Authors:** Geng Chen, Xianjie Xu, Qingtian Zeng, Yu-Dong Zhang

**Affiliations:** 1 College of Electronic and Information Engineering, Shandong University of Science and Technology, 266590 Qingdao, China; 2 School of Computing and Mathematical Sciences, University of Leicester, LE1 7RH Leicester, UK

**Keywords:** Internet of vehicles, Computation offloading, Incentive mechanism, Proximal policy optimization(PPO), Network utility, Task processing delay

## Abstract

With the continuous development of the Internet of Vehicles(IoV), Vehicle Edge Computing(VEC) has become a key technology for computational resource scheduling, but more and more smart devices are connected to the internet, which makes it difficult for traditional Vehicle Edge Networks(VEN) to deal with tasks in time. In this paper, in order to cope with the challenges of the large number of devices accessing the internet, we propose a vehicle-assisted computation offloading algorithm based on proximal policy optimization(VCOPPO) for User Equipment(UE) tasks, and it combines dynamic parked vehicles incentives mechanism and computational resource allocation strategy by using road vehicles and parked vehicles as edge servers. Firstly, a non-convex optimization problem combining VEN utility and task processing delay is formulated, subject to the constraints of the residual energy and the transmission rate of the task. Secondly, the proposed VCOPPO is used to solve the formulated non-convex optimization problem, and we use stochastic policy to obtain the optimal computation offloading decisions and resource allocation schemes. Finally, the experimental results have shown that the proposed VCOPPO has an excellent performance in network reward and task processing delay respectively, and it can effectively schedule and allocate computational resources. Compared with using Dueling Deep Q Network(Dueling DQN), Deep Q Network(DQN) and Q-learning methods, the proposed VCOPPO improves the network reward by 31%, 18% and 91%, reduces the delay in task processing by 78%, 63% and 74%, respectively.

## Introduction

The arrival of the 5G era has greatly promoted the development of the IoT era and opened the door to new network architectures and intelligent services [1], along with more and more intelligent devices connected to the internet, a large number of sensors and information from device terminals will be handed over to the network center for processing [2], and this information needs not only timely processing but also intelligent data analysis, at this time, traditional base stations and cloud computing can no longer meet the needs of users due to they will have higher latency and limited computational power. The concept of edge computing has become a key technology to solve this problem, because in this era, a large amount of data in our daily life will be generated at the edge of the network, and many applications will be deployed at the edge of the network to use these data. Currently, more than half of the IoT devices that generate data will be stored, processed, and analyzed at the edge of the network. Edge computing means that data can be processed at the edge of the network, here the edge is defined as the network resource on the path between the data source and the network center, which can reduce task response time, improve task processing efficiency and reduce the pressure on the network center. [3, 4].

By using the concept of edge computing we can offload tasks to other places with computational resources. In the era of highly developed IoT, there will be many smart devices that have some task processing capabilities themselves, such as vehicles, Unmanned Aerial Vehicles(UAV), etc., we can use them when these computational resources are idle, and the tasks can be offloaded to these places with idle resources, thus providing latency-sensitive applications with low latency, mobility, and location-aware support, reducing the pressure on the network center and improving the user’s sense of online experience [5, 6]. However, computation offloading needs to be considered in various aspects, such as offloading should be done dynamically in each computation way and it needs to determine which ways are worth offloading [7], and how to allocate the bandwidth and reduce the response time of the application for computationally intensive applications, and it needs to allocate the communication and computational resources of each node according to the state of the system [8, 9]. If mobile devices are used as edge servers, it is also necessary to consider the mobility of the devices, the distribution of the devices, and the collaboration between the devices [10, 11], so how to perform reasonable computational offloading is a problem worth studying.

In this paper, we propose a vehicle-assisted computation offloading algorithm based on proximal policy optimization(VCOPPO) for the optimization problem combining Vehicle Edge Network(VEN) utility and task processing delay. The main contributions of this paper are as follows.

1. Firstly, We consider a novel scenario using road vehicles and parked vehicles as edge servers, these two types of vehicles will provide computational resources to the User Equipment(UE).
2. Secondly, combining dynamic parked vehicle incentives and computational resource allocation strategies with solutions to problems posed by vehicle mobility, a non-convex optimization problem is formulated that combines VEN utility and task processing delay. An optimal decision is obtained by maximizing this optimization problem.
3. Then, the proposed VCOPPO is used to solve a non-convex optimization problem with the objective function, and we choose the optimal offloading decision and resource allocation policy by stochastic policy.
4. Finally, we conducted experiments and compared them with algorithms based on the other four methods, which demonstrated the excellent performance of VCOPPO in terms of network reward and task processing delay, respectively.

The layout of the rest of the paper is shown below. Section 2 presents related work, Section 3 describes the scenario and the objective function combining VEN utility and task processing delay, Section 4 introduces the proposed VCOPPO to solve a non-convex optimization problem, then the experimental results based on the five methods are described in Section 5, and finally, we conclude in Section 6.

## Related Work

The combination of computation offloading and machine learning has brought new possibilities for our research, but there are some challenges in its implementation [12], such as how to dynamically offload tasks from devices with insufficient computational resources to edge servers, while minimizing the average task completion delay, how to handle task conflicts when multiple users need to be offloaded at the same time [13]. In order to solve these problems, firstly, a new type of network architecture needs to be built, in the era of IoT, multiple smart devices are equipped with computational resources, their resources should be utilized to relieve our computing pressure, so we can build a new architecture combining vehicular and operator network, to study the integrated dynamic link utilization in edge networks [14, 15]. Combine vehicles to build a new architecture that can dynamically coordinate edge computing and caching resources, which can improve the system utilization [16]. If vehicles are used as carriers, the movement of vehicles will bring some problems, [17]proposed the idea that application migration can be used to solve the delivery problem of tasks brought by the mobility of vehicles while facing the fast traffic flow on highways, [18] gave a way to communicate between vehicles by means of dynamic cluster communication using spatial reusability. In addition, for some complex terrain obstructed by dense buildings and other complex terrains, the UAV can also be used as a relay node, using the flexibility of the UAV to assist the vehicle in offloading computing tasks [19, 20].

After constructing a reasonable architecture, a performance index needs to be found to judge the merits of the system so that the final optimization function can be derived, in which performance indexes such as network utility, task processing delay, and energy consumption are the hotspots of research. From the perspective of network managers [21–23] establishing a charging and spending model for communicating and computing, focusing on maximizing the long-term utility of the edge network, and treating the task processing delay as a constraint, on the basis of which derive the objective function and then use optimization methods to solve it, while focusing on the revenue, we also need to pay attention to the energy consumption of the system and the user’s internet access delay problem. For the case that the time-varying characteristics of vehicles make it difficult to perform effective offloading under the energy constraint, [24] gives an objective function for minimizing the total task delay under the long-term energy constraint, and then proposes an online multi-decision scheme to solve the specified objective function. In addition, we can combine multiple indexes to build the objective function to meet the requirements of different occasions.

There is still an important issue how to solve the optimization problem of the optimization function after the function is obtained above using various indexes. The various optimization algorithms for solving the optimization function are very worthy directions to study, and using different optimization algorithms may produce different results. Most of the final functions obtained according to some constraints are non-convex problems and difficult to solve directly. Some traditional algorithms are used to solve it, [25] defines the combination of delay and energy consumption as the objective function, the communication is modeled by a genetic algorithm, and the strategy corresponds to the chromosomes in the algorithm, finally, an efficient offloading is achieved. The transaction process of computing data between UAVs and vehicles can also be modeled as a bargaining game to obtain the optimal policy [26], while in [27], an accelerated gradient algorithm that jointly optimizes the offloading rate and transmission time is proposed, which improves the convergence speed compared to traditional algorithms and finds the optimal value at a faster rate. In addition, for the multi-objective offloading problem, [28] proposed an improved decomposition-based multi-objective evolutionary algorithm that uses a delay-based execution position initialization method for the population initialization problem, and the results show that it outperforms the heuristic algorithm in terms of convergence and diversity of the obtained non-dominated solutions. For intensive task offloading, [29] used a Lyapunov-based elastic parallel search algorithm to solve the proposed energy and delay-based optimization objectives.

In addition to the classical traditional algorithms, the rapid development of machine learning in recent years has given us new tools that make data processing easier and more efficient, among them, Deep Reinforcement Learning is often used to solve non-convex optimization problems, which includes value-based and policy-based methods. The Q-learning, DQN, and Dueling DQN are all value-based deep reinforcement models that can formulate the dynamic offloading problem as a Markov decision process, using DQN to approximate the objective value function to obtain an optimized offloading policy, even for multi-user mobile edge computing networks, DQN-based algorithms have advantages over traditional algorithms [30–32]. DQN may suffer from overestimation due to choosing the maximum action value each time in order to avoid this situation, Double DQN are generated, in [33], delay constraints and uncertain resource requirements of heterogeneous computing tasks, and in order to avoid dimensional disasters and overestimation using DDQN-based algorithms, which eventually proved the effectiveness of the algorithm. It is also possible to change the network structure of DQN to produce Dueling DQN, [34] firstly preprocesses the data and then uses Dueling DQN for optimization of the objective function, its convergence speed is faster than DQN. However, when faced with a high-dimensional or continuous action space, policy-based algorithms are more efficient and easier to reach convergence, and in the face of the state space explosion problem in distributed mobile fog computing, DDPG can be used to solve the optimization problem of the objective in a high-dimensional state space, and also its network mechanism can be combined with Graph Convolutional Networks (GCN), thus achieving improved performance [35, 36]. The Proximal Policy Optimization(PPO) algorithm used in this paper, as the most widely applicable algorithm, has an excellent performance in either discrete or continuous state-action spaces, [37] using PPO to solve the optimal objective factor in a reasonable state-action space, obtain accurate continuous actions, and achieve fast convergence.

By reading the above literature, they can all relieve the computational pressure of the network center using the computational resources at the edge of the network, but the scenarios of most literature are too single, and only the resources of the vehicles on the road are studied, which cannot serve the users well when there are fewer resources of vehicles on the road such as weekends, so it cannot fit the actual situation better. In the era of the Internet of Vehicles, vehicles will be installed with computation and communication units, the time spent in the parking lot is more than the time spent on the road for each vehicle, so the computational resources when the vehicle is idle can also be used. Besides, we need to schedule and allocate the computational resources of each computation way reasonably according to the system state to meet the needs of different types of tasks, and our work will be described in detail below.

## System Model

**Fig. 1:**
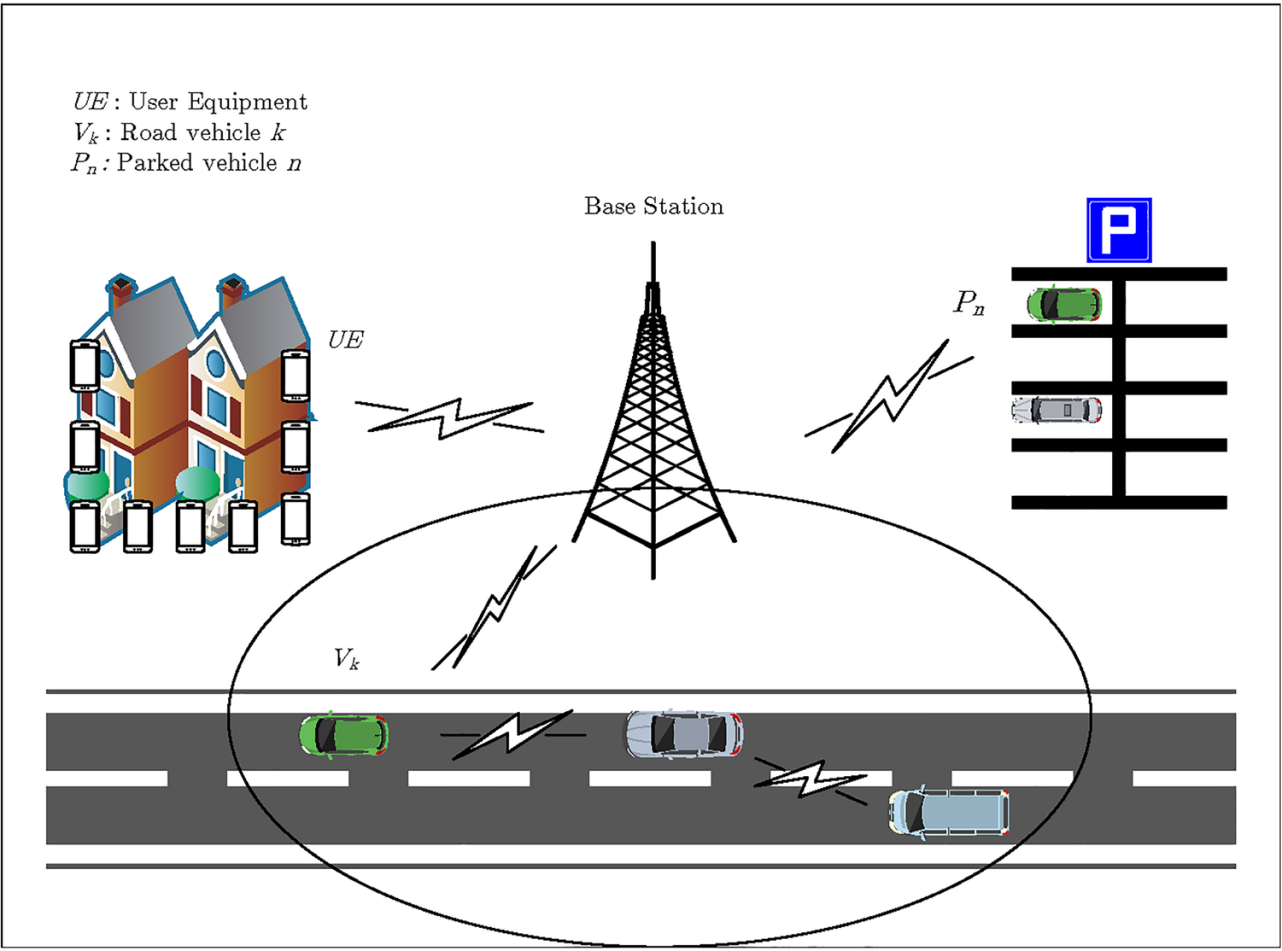
Vehicle-assisted offloading network

As shown in Fig. 1, this paper considers a cell near the road, and a parking lot with an incentive mechanism near the cell. Tasks will be sent to the base station for processing when the user *UE*$_{i}$ in the cell has a computation task generated. The base station makes decisions based on maximizing the current objective function. There are three ways to compute, the first one is the base station for local computing, we set the energy range and resource allocation strategy for the base station, the computational power changes with the remaining energy and resource allocation strategy. The result will be returned to the user after the computation is completed; The second one is the tasks are offloaded to the vehicle *V*$_{k}$ on the road, the vehicle will also change its computational power according to the remaining energy and resource allocation strategy. At this time, the vehicle *V*$_{k}$ will be selected within the coverage range of the base station for offloading, if the vehicle fails to complete this task within the coverage range as the vehicle moves, the application migration will be combined before the vehicle drives out of the range, the remaining tasks are packaged and sent to the vehicle behind for processing and returned to the user through the base station after the calculation is completed; The third one is the tasks are offloaded to the vehicle *P*$_{n}$ in the parking, the computational power of the parked vehicles will change with the number of vehicles, the remaining energy, and the resource allocation strategy. The dynamic incentive mechanism of the parked vehicle will attract the vehicle owners to park their vehicles here, thus it expands the computational resources of the cell. In addition, each scheme sets dependencies, an error message will be returned for redecision if the remaining energy of such scheme is insufficient.

For the problem caused by the mobility of vehicles. The application migration can be combined to solve it, we assume that the computation tasks are generated by a specific application. As shown in Fig. 1, the base station has a coverage area, if the first vehicle is performing the computation tasks, as the vehicle moves, the first vehicle is about to drive out of the coverage area of the base station, and the remaining tasks will be stored and packaged to sent to the vehicle behind if the computation tasks are not completed, the vehicle behind will install this packet after receiving the data and then has the ability to continue processing the task so that the problem caused by vehicle mobility should be improved.

For the dynamic parked vehicles incentive mechanism. The vehicle will have communication and computation units in the era of connected cars, most vehicles spend more time parked in the parking than on the road, so the computational resources of the vehicles in the parking should also be utilized. We will give rewards to the vehicles in the parking lot for participating in the task in order to attract more owners to park here. The agent of the VCOPPO proposed in this paper dynamically selects different reward unit prices depending on the system state, and a parameter is used to represent the relationship between the reward unit price of the parked vehicles and the number of parked vehicles. Furthermore, the agent will choose the larger unit price if the number of tasks in the VEN is large and there are not enough remaining computational resources to process all the tasks, and there will be more parked vehicles involved in the task processing if the agent chooses a larger unit price, so that VEN can cope with all the tasks well due to the increase of computational resources in the parking lot. The rewards for the moving vehicles are not considered in this paper, because the moving vehicles need to constantly communicate with the network in the future era of connected cars, and we set up the parked vehicles reward mechanism to attract vehicle owners to park their vehicles here by giving them rewards, but the road vehicles are processing tasks while driving, there is no case of changing the route of vehicles through the reward mechanism.

For the computational resource allocation strategy, in this paper, we equate the remaining computational resources of the three computational ways to the remaining energy of each way, and the allocation of computational resources is equivalent to the allocation of the energy consumed to complete each task. Two energy ranks are set in the action space to determine the energy consumed to complete each task, and the agent can choose between these two energy ranks when making decisions. If the larger value is chosen, the more energy is consumed to complete this task, furthermore, the more computational resources are allocated to this task, resulting in larger computational power for this way; if a smaller value is chosen, the less energy is consumed to complete the task, the less computational resources are allocated to the task, resulting in less computational power for this way.

This section gives the delays of task processing in three ways, which include the time for task communication and computation. The communication time from the users to the base station is omitted, only the time from the base station to each location is calculated. The utilities of the three ways are given which include the communication utility and the computation utility, and the computation utility needs to be subtracted from the return to the parked vehicles. Then, an objective function is set, and we use the proposed VCOPPO to solve the optimization problem of the objective function.

Define *d*$_{i}$ $\in$ {0,1} as the decision whether to offload, *d*$_{i}$ = 1 means the base station performs local computing, *d*$_{i}$ = 0 means offloading, then define *p*$_{i}$ $\in$ {0,1} and *v*$_{i}$ $\in$ {0,1}, *p*$_{i}$ = 1 means the task offloads to parked vehicles, otherwise, *p*$_{i}$ = 0; *v*$_{i}$ = 1 means the task offloads to vehicles on the road, otherwise, *v*$_{i}$ = 0.

### Communication Model

The communication time includes the time from the base station to the road vehicles and the time from the base station to the parking. Here we ignore the communication time from the user to the base station due to the close distance, and we also omit the return time of the task.

1. Base station to road vehicles: We define *P*$_{i}$ as the transmission power, *g*$_{k,i}$ is the channel gain of the wireless propagation from user *i* to road vehicle *k*, $\omega$ is the mutual interference between vehicles, and $\sigma$ is the noise power, the spectrum efficiency of the task *i* to the vehicle *k* on the road can be calculated as

1 $$\begin{aligned} e_{k,i}^{v}= log_{2}\left( 1+\frac{S}{N} \right) =log_{2}\left( 1+\frac{P_{i}g_{k,i}}{\omega +\sigma } \right) \end{aligned}$$

Define $\eta ^{v}_{k,i}$ as the percentage of spectrum allocated to the task by the vehicle of road, the task transmission rate between the road vehicle and the base station can be defined as

2 $$\begin{aligned} R_{k,i}^{v}=\eta ^{v} _{k,i}Be_{k,i}^{v} \end{aligned}$$

where *B* is the bandwidth from the base station to the road vehicles.

(2) Base station to parked vehicles: The spectrum efficiency of the task *i* to the vehicle *n* in the parking can be calculated as

3 $$\begin{aligned} e_{n,i}^{p} =log_{2}\left( 1+\frac{P_{i}g^{p}_{n,i}}{\omega +\sigma ^{p} } \right) \end{aligned}$$

where *P*$_{i}$ is the transmission power, $g^{p}_{n,i}$ is the channel gain of the wireless propagation from task *i* to parked vehicles $n$, $\omega$ the mutual interference between vehicles, and $\sigma ^{p}$ is the noise power. Define $\eta ^{p}_{n,i}$ as the percentage of spectrum allocated to the user by the vehicle, $B_{0}$ is the bandwidth from the base station to the vehicles in the parking lot. The task transmission rate between the parked vehicle and the user can be calculated as

4 $$\begin{aligned} R_{n,i}^{p}=\eta ^{p}_{n,i}B_{0}e_{n,i}^{p} \end{aligned}$$

### Computation Model

The task *i* is defined as $\mathbf {S}_{i}= \{H_{i}, Z_{i}\}$, where *H*$_{i}$ denotes the size of the task data, and *Z*$_{i}$ denotes the number of computational resources required to complete the task. We can choose the energy consumed to process a task among the two energy ranks, the more energy consumed means the stronger the current computational power of the task, and the smaller the computation time of the task. The initial energy value is set for each scheme as Table1, the computational power of each scheme will change according to the amount of energy remaining.

1. Local computinbg: We define $C_{local, i}$ as the computational power of the local base station, the local computing time can be expressed as
  5 $$\begin{aligned} T_{i}^{local}=\frac{Z_{i}}{C^{local}_{i}} \end{aligned}$$
  6 $$\begin{aligned} {C^{local}_{i}= \varphi ^{local}\zeta ^{local} _{i} E^{local}_{i}} \end{aligned}$$
  where $\varphi ^{local}$ is the ratio of computational power to residual energy, $\zeta ^{local} _{i}$ is the influence coefficient of the energy rank by this task on the computational power, $E^{local}_{i}$ is the remaining energy of the base station when processing task *i*. Since the local computing does not require the communication time of the transmission task, it only needs to count the computation time.
2. Computation of road vehicles: The road vehicle needs to transmit the task through the base station, so its total time includes the communication time and the computation time of the task. The communication time can be calculated as
  7 $$\begin{aligned} T_{comm,i}^{v}=\frac{H_{i}}{R_{k,i}^{v}} \end{aligned}$$
  The computation time can be expressed as
  8 $$\begin{aligned} T_{comp,i}^{v}=\frac{Z_{i}}{C^{v}_{k,i}} \end{aligned}$$
  9 $$\begin{aligned} {C^{v}_{k,i}= \varphi ^{v}\zeta ^{v} _{k,i} E^{v}_{i} } \end{aligned}$$
  where $C^{v}_{k, i}$ is the computational power of the vehicle *k* in the road vehicle when processing the task *i*, which is the computational power of one vehicle as we assume that only one road vehicle can participate in the computation, the task will be sent to the next vehicle to continue the computation when the vehicle is about to leave the coverage area of the base station. $\varphi ^{v}$ is the ratio of computational power to residual energy, and $\zeta ^{v} _{k, i}$ is the influence coefficient of the energy rank by this task on the computational power, $E^{v}_{i}$ is the residual energy of the vehicles on the road. The total time required for the vehicle on the road to complete the computation task from the communication time plus the computation time as
  10 $$\begin{aligned} T_{i}^{v}=T_{comm,i}^{v}+T_{comp,i}^{v} \end{aligned}$$
3. Computation of parked vehicles: We assume that the larger the reward given to the owner, the more vehicles will be in the parking. The vehicles in the parking can jointly calculate the task, and the base station also needs to transmit the task first and then calculate it by the vehicles. Its processing delay also includes communication time and computation time, the communication time is expressed as
  11 $$\begin{aligned} T_{comm,i}^{p}=\frac{H_{i}}{R^{p}_{n,i}} \end{aligned}$$

The computation time is calculated as

12 $$\begin{aligned} T^{p}_{comp,i}=\frac{Z_{i}}{C^{p}_{i}} \end{aligned}$$

13 $$\begin{aligned} {C^{p}_{i}}= \varphi ^{p}\zeta ^{p} _{i} E^{p}_{i}N_{i} \end{aligned}$$

We assume that the task is communicated by vehicle n, the vehicles in the parking can jointly calculate the task and each vehicle has the same computational power. Where $C^{p}_{i}$ is the common computational power for parked vehicles when processing the task *i*, $\varphi ^{p}$ is the ratio of computational power to residual energy, and $\zeta ^{p} _{i}$ represents the influence coefficient of the energy rank assigned by the task on the computing power of all vehicles in the parking lot, $E^{p}_{i}$ is the common residual energy of the vehicles in the parking, *N*$_{i}$ represents the number of vehicles involved in the calculation in the parking when task *i* is calculated. We assume that the parameter of the number of vehicles changing with the reward $r^{p}_{i}$ for the owner is $\xi$, and the change in the number of vehicles and the unit price given to them can be expressed as

14 $$\begin{aligned} N_{i}=\xi r^{p}_{i} \end{aligned}$$

Then the total time required for the parked vehicles to complete this task is given by

15 $$\begin{aligned} T_{i}^{p}=T_{comm,i}^{p}+T_{comp,i}^{p} \end{aligned}$$

### VEN Utility

The VEN utility includes VEN charging the user for task processing and the output of VEN, where local computing has only computing utility since it does not need to transmit tasks, and two types of vehicle computing include communication utility and computation utility because it needs to transmit tasks first and then compute. We assume that the VEN has no output in the local computing way, and the output in the vehicle computing ways includes the spectrum resources leased by VEN in the wireless network and the rewards given to the parked vehicles. We uniformly express the VEN utility as follows.

1. Communication Utility: VEN needs to charge users for transmitting tasks with road vehicles and parked vehicles, assuming a unit price $\alpha _{i}$, and then the VEN needs to lease spectrum from the wireless network for communicating with both road vehicles and parked vehicles, assuming their unit prices are $\beta ^{v}_{k, i}$, $\beta ^{p}_{n, i}$. The VEN communication utility is 0 when the local computing is selected, so the communication utility in the three ways is calculated as
  16 $$\begin{aligned} F_{comm,i}=(1-d_{i})(\alpha _{i}-v_{i}\beta ^{v}_{k,i}-p_{i}\beta ^{p}_{n,i})H_{i} \end{aligned}$$
2. Computation Utility: The unit price of the computational task $S_{i}$ charged by the vehicle edge network to the user is $b_{i}$, while the unit price of the resources rented by the vehicle edge network to the wireless network is $\varepsilon ^{v}_{k, i}$, $\varepsilon ^{p}_{n, i}$, respectively. In order to attract vehicle owners to park their vehicles here, VEN also needs to give a reward to the vehicle owner, defining its unit price as $r^{p}_{i}$, thus the following formula for computation utility is obtained by
  17 $$\begin{aligned} F_{comp,i}=(b_{i}-v_{i}\varepsilon ^{v}_{k,i}-p_{i}(\varepsilon ^{p}_{n,i}+r^{p}_{i}))Z_{i} \end{aligned}$$
  So the total VEN utility by adding the communication utility and the computation utility is expressed as
  18 $$\begin{aligned} F_{u}(i)=F_{comm,i}+F_{comp,i} \end{aligned}$$

### Problem Formulation

We have known the scenario and the basic model of this paper from the above introduction, then the objective function of this paper by combining network utility and task processing delay is formulated, and we select the optimal offloading decisions by comparing the objective function of three ways.

The objective function in this paper takes the form of the difference between network utility and task processing delay generated by completing all tasks, where the difference between network utility and delay generated by completing task *i* is shown below

19 $$\begin{aligned} F(i)=F_{u}(i)-\phi T_{i} \end{aligned}$$

where $F_{u}(i)$ denotes the network utility generated by the transmission and calculation of task *i*, $\phi$ denotes the weight of the delay of task processing, $T_{i}$ denotes the time required to calculate this task, we can adjust $\phi$ to change the focus to suit different occasions. The optimization problem of this paper is formulated by summing up the difference in completing each task as follows

$$\begin{aligned} \underset{\underset{\eta ^{v} _{k,i},\eta ^{p}_{n,i},\varphi ^{local},\varphi ^{v},\varphi ^{p}}{d_i,v_i,p_i}}{\max }\sum _{i=1}^{I}F(i) \end{aligned}$$

**s.t.**

20 $$\begin{aligned} c1:d_{i}+v_{i}+p_{i}=1\nonumber \\ c2:d_{i},v_{i}, p_{i}\in \left\{ 0,1 \right\} \nonumber \\ c3:\eta ^{v} _{k,i}, \eta ^{p}_{n,i}\in (0,1)\nonumber \\ c4:E^{local}_{i},E^{v}_{i},E^{p}_{i}\ge E^{rank}_{min} \end{aligned}$$

where *I* is the number of tasks sent at a time, and our goal is to maximize the objective function of the tasks sent at a time, condition (c1),(c2) ensures that each task can only choose one of the three ways, condition (c3) ensures that the bandwidth of road vehicles and parked vehicles communicating with the base station cannot exceed its total bandwidth, condition (c4) ensures that the residual energy for each mode is not less than the minimum energy rank when processing tasks. The purpose of setting this objective function is to balance the network utility and task processing delay, the larger the objective function, the greater the difference between network utility and task processing delay, that is, the relatively bigger network utility and the smaller task processing delay, which is the best state we pursue.

The computational power of the three schemes and the reward given to parked vehicles are dynamically changing, therefore, it is necessary to collect a large number of network states and make decisions based on the states, the proposed VCOPPO is used to solve this problem in this paper.

## The Proposed Vehicle-assisted Computation Offloading Algorithm Based on Proximal Policy Optimization(VCOPPO)

We approximate the decision process in this paper as a Markov process, the action at the next moment is only related to the state at the current moment, as described below, and the Markov decision process can be solved using the proposed VCOPPO.

### Algorithm Principle of PPO

Reinforcement learning is to influence the environment by the agent, the environment makes corresponding changes according to the action and feeds back to the agent, and then the agent selects the next action according to the current state. Reinforcement learning is divided into value-based and policy-based, where the value-based algorithm is to select the action with the highest score by calculating the score of the action, for example, the comparison methods Q-learning, DQN, and Dueling DQN in this paper, while we use another policy-based method in this paper, PPO, which uses some kind of policy to select actions based on the determination of the value function. In the discrete action space of this paper, an action is selected by the observed information for reverse transmission, and then the probability of the selected action is enhanced and weakened according to the network rewards directly, where the probability of good action being selected is increased and bad actions is weakened in the next choice. The training of the algorithm alternates between sampling data interactively with the environment and optimizing an alternative objective function using stochastic gradient ascent.

1. Clipped Surrogate Objective: PPO algorithm is a new type of Policy Gradient algorithm, Policy Gradient algorithm is very sensitive to the step size, but it is difficult to choose the appropriate step size, and the difference between the change of old and new policy during training is not conducive to learning if it is too large. In order to solve these problems, PPO uses the Actor-Critic structure to estimate the V-value using the critic network, so as to achieve a single-step update and uses importance sampling to sample more data with a fixed policy different from the current policy and use it repeatedly. The expression of the update gradient of the policy gradient is written as

21 $$\begin{aligned} \widehat{g}=\widehat{E}_{(s_{t},a_{t})\sim \pi _{\theta }}\left[ \bigtriangledown _{\theta }log\pi _{\theta }(a_{t}|s_{t})\widehat{A}^{\theta }_{t} \right] \end{aligned}$$

where $\pi _{\theta }$ is the strategy with parameter $\theta$, and $\widehat{A}^{\theta }_{t}$ is the estimate of moment *t* for the dominance function, which is the dominance function, indicating the advantage obtained by taking action *a* in state *s* at this time, and $\widehat{E}_{(s_{t},a_{t})\sim \pi _{\theta }}$ indicates the average value over the sample. The algorithm usually constructs an objective function with a gradient equal to the gradient of the sub-strategy when performing the gradient update, and then performs gradient ascent for this objective function, where the objective function is given as

22 $$\begin{aligned} L^{PG}(\theta )=\widehat{E}_{(s_{t},a_{t})\sim \pi _{\theta }}\left[ log\pi _{\theta }(a_{t}|s_{t}\widehat{A}^{\theta }_{t}) \right] \end{aligned}$$

The importance sampling here is like we need to sample *x* from the distribution *p* and then calculate the expected value after bringing in *f*(*x*). If we have no way to sample the data in the distribution *p*, we can sample *x* from another distribution *q*, as follows.

23 $$\begin{aligned} \int f(x)p(x)dx=\int f(x)\frac{p(x)}{q(x)}q(x)dx=E_{x\sim q}\left[ f(x)\frac{p(x)}{q(x)}\right] \end{aligned}$$

where *p*(*x*)/*q*(*x*) becomes the importance weight to correct the difference between these two distributions, we transform the expectation of *x* sampled from *p* into the expectation of *x* sampled from *q*. The policy gradient and objective functions after importance sampling are shown below, respectively.

24 $$\begin{aligned} \widehat{g}^{'}=\widehat{E}_{(s_{t},a_{t})\sim \pi _{\theta ^{'}}}\left[ \frac{\pi _{\theta }(a_{t}|s_{t})}{\pi _{\theta ^{'} }(a_{t}|s_{t})}\bigtriangledown _{\theta }log\pi _{\theta }(a_{t}|s_{t})\widehat{A}^{\theta ^{'}}_{t} \right] \end{aligned}$$

25 $$\begin{aligned} L^{'PG}(\theta )=\widehat{E}_{(s_{t},a_{t})\sim \pi _{\theta ^{'}}}\left[ \frac{\pi _{\theta }(a_{t}|s_{t})}{\pi _{\theta ^{'} }(a_{t}|s_{t})} \widehat{A}^{\theta ^{'}}_{t}\right] \end{aligned}$$

where $\theta$ is the target strategy and $\theta ^{'}$ is the behavioral strategy, it can be seen that the samples when estimating the strategy gradient are sampled from the behavioral strategy $\theta ^{'}$, and $\widehat{A}^{\theta ^{'}}_{t}$ is also estimated from the behavioral strategy $\theta ^{'}$. If this proxy target function is updated directly, it will result in too much difference in $\frac{\pi _{\theta }(a_{t}|s_{t})}{\pi _{\theta ^{'} }(a_{t}|s_{t})}$, We have chosen the Clipped Surrogate Objective approach to avoid this situation, as follows.

26 $$\begin{aligned} L(s,a,\theta _{k},\theta )= & {} min\left( \frac{\pi _{\theta }(a|s)}{\pi _{\theta _{k} }(a|s)} A^{\pi _{\theta _{k}}}(s,a),\right. \nonumber \\&\left. clip\left( \frac{\pi _{\theta }(a|s)}{\pi _{\theta _{k} }(a|s)},1-\varepsilon ,1+\varepsilon \right) A^{\pi _{\theta _{k}}}(s,a)\right) . \end{aligned}$$

where $\varepsilon$ is a parameter to measure the degree of deviation between the new strategy and the old strategy, when $A^{\pi _{\theta _{k}}}(s,a)>0$, that is, the advantage function is positive, increasing the possibility of the corresponding action appearing $\pi _{\theta _{k}}(a|s)$, the value of the objective function can be increased, once $\pi _{\theta _{k}}(a|s)$ exceeds $1 + \varepsilon$, the function will be truncated to $(1+\varepsilon )A^{\pi _{\theta _{k}}}(s,a)$, at this time $\pi _{\theta _{k}}$ and then increase is useless. Similarly, when $A^{\pi _{\theta _{k}}}(s,a)<0$, the possibility of the corresponding action appearing $\pi _{\theta _{k}}$ need to be reduced, the value of the objective function can be increased , once $\pi _{\theta _{k}}$ is less than $1 - \varepsilon$, the objective function will be truncated to $1 - \varepsilon$, at this time $\pi _{\theta _{k}}$ will not be reduced. This is to prevent the situation where $\frac{\pi _{\theta }(a|s)}{\pi _{\theta _{k}}(a|s)}$ are too different.

(2) Generalized Advantage Estimation(GAE): The GAE method is used in PPO to estimate the advantage function, which is shown in the following equation

27 $$\begin{aligned} A^{GAE(\delta ,\lambda )}_{t}=\sum \limits _{l=0}^{\infty }(\delta \lambda )^{l}[R_{t+l}+\delta V(s_{t+l+1})-V(s_{t+1})] \end{aligned}$$

where *V* is the state value function and $\delta$ is the weight value, GAE takes into account the advantage of each subsequent moment of state *s*, respectively, and then weights and sums according to the distance from the current state.

### The proposed VCOPPO

The proposed VCOPPO is shown in Algorithm 1, where the state, actions, and reward functions are shown below. States have five elements in this paper, including the number of completed tasks, the number of vehicles, and the current energy remaining in each of the three ways.

28 $$\begin{aligned} states=[ctasks, N_{i}, E^{lacal}_{i}, E^{v}_{i}, E^{p}_{i}] \end{aligned}$$

States have five elements in this paper, where *ctask* indicates the number of completed tasks, $N_{i}$ indicates the number of vehicles, $E^{lacal}_{i}$ represents the remaining energy of local, $E^{v}_{i}$ represents the remaining energy of road vehicles, $E^{p}_{i}$ represents the common residual energy of parked vehicles.

29 $$\begin{aligned} actions=[W_{i},r^{p}_{i},E^{rank}_{i}] \end{aligned}$$

where $W_{i}$ denotes the offloading schemes in three ways including local, road vehicle, and the parked vehicle. $r^{p}_{i}$ denotes the unit price of the reward given to the owner, we define a range of 0.1 - 0.5 for this unit price, furthermore, the agent can choose an incentive unit price among 0.1, 0.2, 0.3, 0.4, 0.5 for each decision, the agent can choose a different unit price in this range, $E^{rank}_{i}$ denotes the energy rank of the processing task. There are two energy ranks that represent the energy consumption value to complete this task, the agent can choose different energy ranks according to the task and the number of vehicles. Further, the energy value corresponding to the energy rank selected by the agent in each computation way will be deducted at the completion of each task.

30 $$\begin{aligned} reward=\sum \limits _{i\in I}^{}F(i) \end{aligned}$$

The network reward function in this paper is the objective function of the system, which represents the difference between the network utility of completing the whole task and the delay, and a negative reward is given when the residual energy for a certain decision is chosen to be insufficient. In this paper, we use the proposed VCOPPO to improve or reduce the possibility of selected behaviors by updating the network parameters continuously. The desired action is outputted in a certain state after learning for a period of time.

**Algorithm 1:**
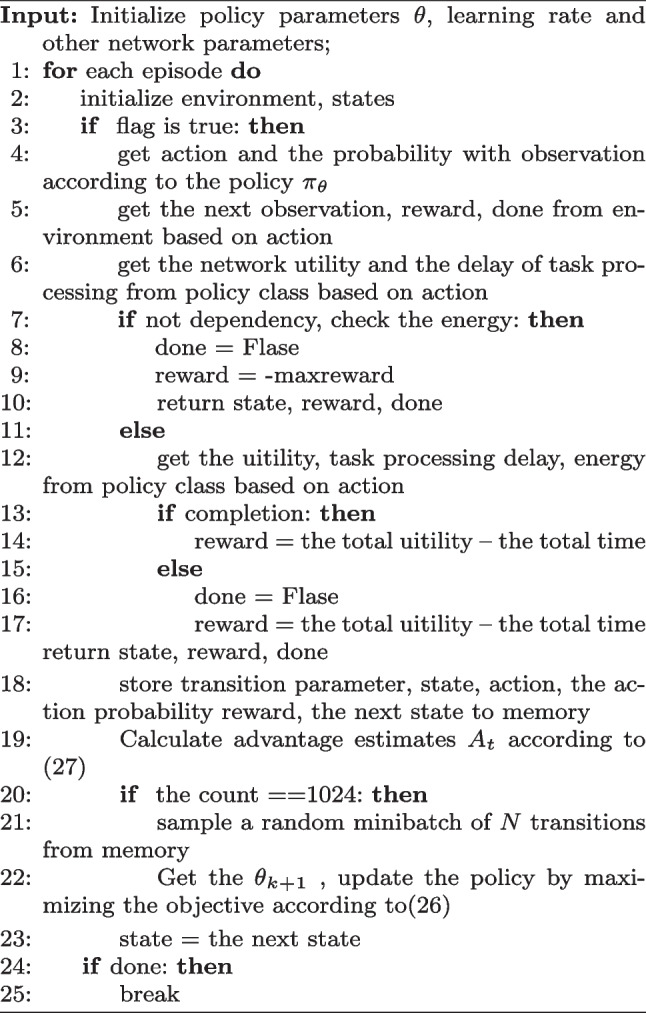
The proposed VCOPPO

## Experimental Results and Analysis

To demonstrate the performance of the proposed scenario and method, we consider a $5\times 5\ km^{2}$ cell close to a road, which contains a base station, several moving vehicles, and nearby parking with an incentive mechanism. We assume that the data size and required computing resources of each task are fixed when tasks are created, and the number of tasks can be changed to test the effect of the different number of tasks on the performance of the algorithm in the experiment. The other parameters as shown in Table 1.

**Table 1:** Simulation parameters table

B	5 MHZ	The bandwidth between base station and road vehicles
$B_0$	8 MHZ	The bandwidth between base station and parked vehicles
$P_{i}$	100 mw	Transmission power of the base station
$\sigma$,$\sigma ^{p}$	-100 dbm	The noise power of road vehicles and parked vehicles
$H_i$	2 M	The size of task
$Z_i$	100 Megacycles	The resources required of task
$\xi$	10	Parameter of the number of vehicles changing with the reward for vehicles
$\phi$	1	The weight of time in the objective function
$E^{rank}$	$\{200,400\}$	Energy rank
$\varphi ^{local},\varphi ^{v},\varphi ^{p}$	1000,1666,1250	Influence factor of computational power and residual energy
$\zeta ^{local}_{i},\zeta ^{v}_{k,i},\zeta ^{p}_{n,i}$	$\{1,2\}$	Influence factor of computational power and energy rank
$E^{local}_{init}, E^{v}_{init,k}, E^{p}_{init,n}$	2100, 500, 500	Initial energy values in base stataion, road vehicles and parked vehicles

In this paper, we model the proposed scenario and solve the formulated non-convex optimization problem using VCOPPO and compare the performance with Dueling DQN, DQN, Q-learning, and Random methods. In VCOPPO, we set the parameters such as mini-batch size = 128, clip = 0.2, discount factor = 0.99, and learning rate = 0.0003. In GAE, we set $\lambda$ = 0.98, learning rate = 0.0003, etc. In both Dueling DQN and DQN methods, we set memory size = 2000, the reward discount = 0.9, greedy = 0.9, batch size = 32. In the Q-learning method, we set greedy = 0.9 and reward decay = 0.9. In the Random method, a random selection of actions is performed in the action space in each decision.

**Fig. 2:**
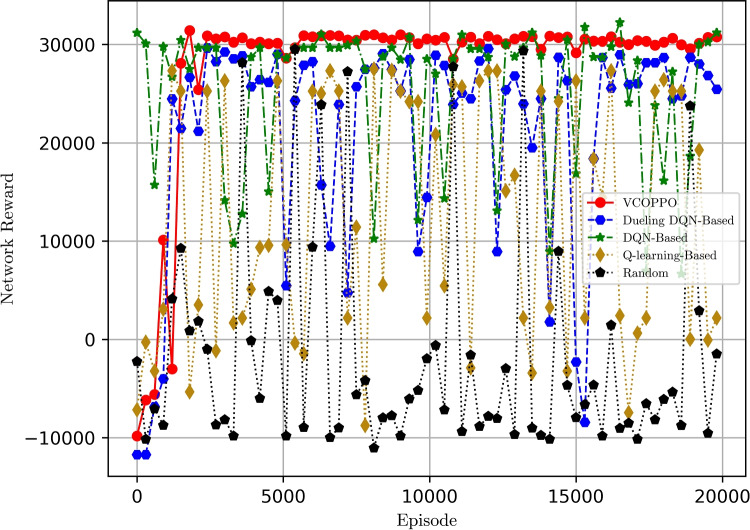
Comparison of network rewards with episodes

Figure 2 shows the comparison of the network reward under five methods when sending ten tasks, it can be seen that the proposed VCOPPO reaches convergence around 2000 times and converge to a high reward among all the methods. This is because the proposed VCOPPO can achieve small batches of updates at multiple training steps by importance sampling, so it uses the optimal offloading policies and resource allocation for the vehicle edge network at each decision. In contrast, most of the rewards of the Random method are negative because it selects the action randomly. Meanwhile, the Q-learning method has a large variation in the reward because it selects the action with the largest value by looking up the table, which can not handle the high dimensional computation well. However, the Dueling DQN method and DQN method have a higher overall reward value than Q-learning due to the addition of neural networks, but there is no convergence, and some large values of DQN maybe because overestimation phenomena have occurred. The results show that the proposed VCOPPO improves network reward by 31%, 18%, and 91%, compared to Dueling DQN, DQN, and Q-learning methods, respectively.

**Fig. 3:**
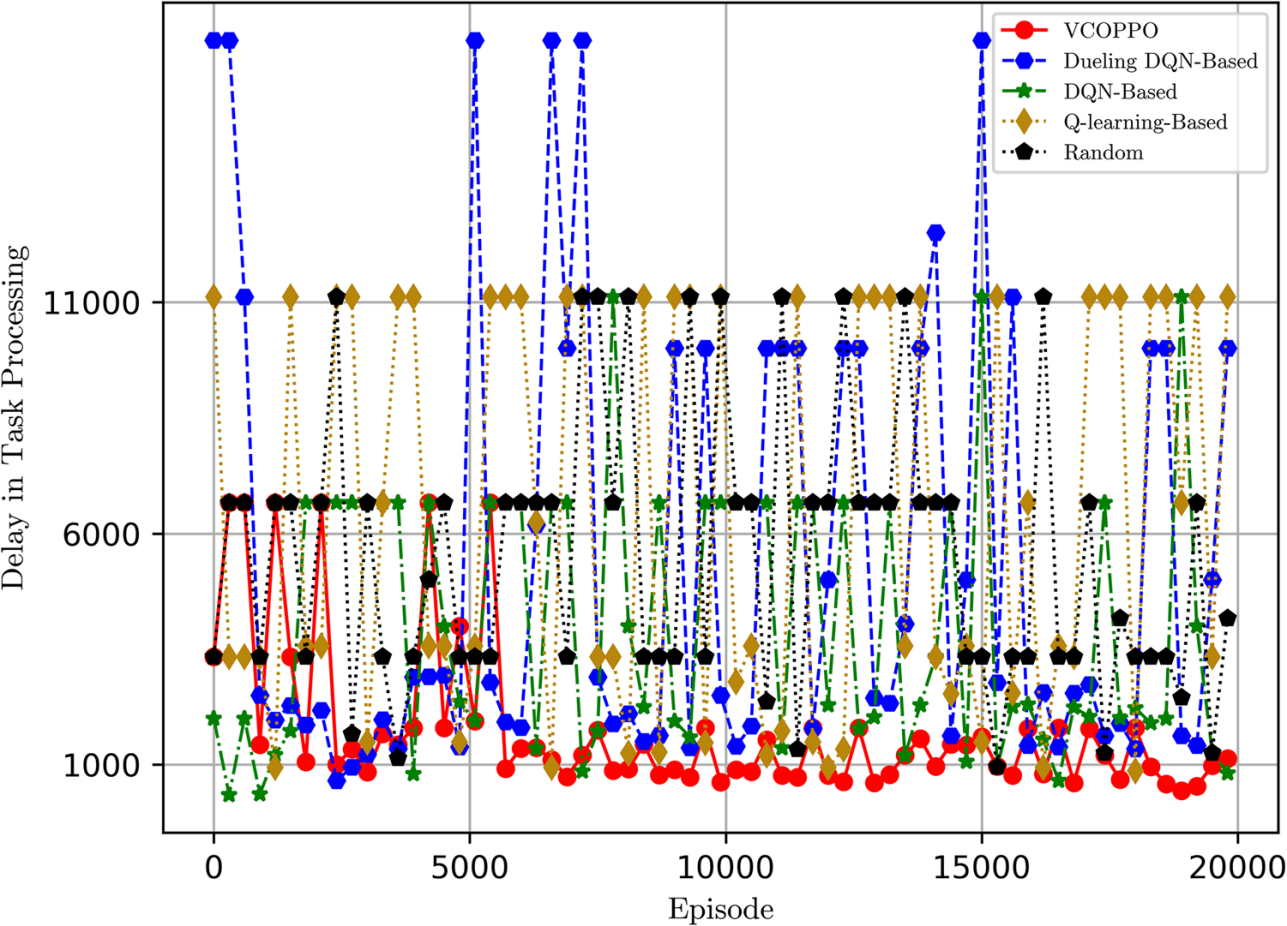
Comparison of delay with episodes

In Fig. 3, we also compare the total processing delay by sending ten tasks, in this paper, we will choose to multiply the maximum delay of the previous task processing by the number of tasks if we encounter constraints such as insufficient energy, so there are some larger values. we can observe that the proposed PPO-based method has the lowest delay among other solutions, and which converges to about 1000 in 6000 times. That is, the proposed VCOPPO always makes the better choice of offloading strategy according to the variable network environment. It is interesting to see that the delay of Random method is not the largest imaginable because it encounters constraints that do not meet the energy requirements in the early stages of task processing, so the maximum delay of previous task processing is also a relatively small value. The Dueling DQN method and DQN method have better task processing delay than Q-learning method but higher than the proposed VCOPPO. This is because neural networks can handle relatively high-dimensional state-action spaces but the value-based approach makes it difficult to converge. The Dueling DQN and DQN methods have some larger task processing delays in particular because of the mechanism of random exploration. The results show that the proposed VCOPPO reduces the delay of task processing by 78%, 63%, 74%, and 76%, compared to Dueling DQN, DQN, Q-learning, and Random methods, respectively.

**Fig. 4:**
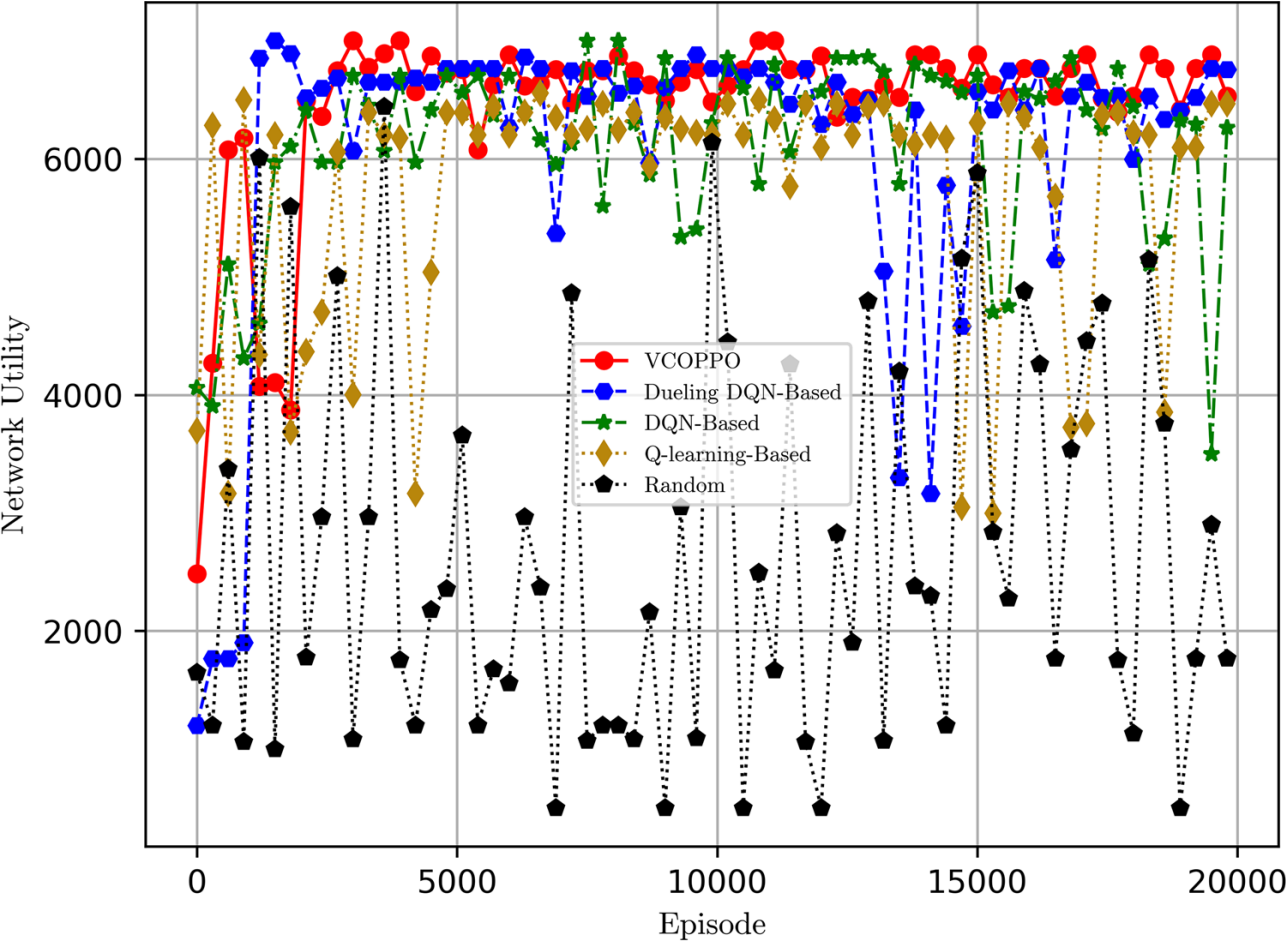
Comparison of network utility with episodes

Figure 4 shows the variation of network utility under the five methods. It can be seen that the proposed VCOPPO does not have a significant advantage over the Dueling DQN, DQN, and Q-learning methods in terms of network utility, because our network reward is set as the difference between the network utility and the task processing delay, and the agent maximizes this difference depending on the state at this time. Combined with Fig. 3, it can be concluded that the proposed VCOPPO has a significant advantage over the Dueling DQN, DQN, and Q-learning methods in terms of task processing delay with the same network utility, due to the fact that the network utility is mainly determined by the computation way assigned to the task, and the proposed VCOPPO, Dueling DQN, DQN and Q-learning can choose the correct computation way after training. However, the delay is mainly determined by the reasonable resource allocation and scheduling, therefore, the proposed VCOPPO can reasonably allocate and schedule computing resources after selecting the correct computing way.

**Fig. 5:**
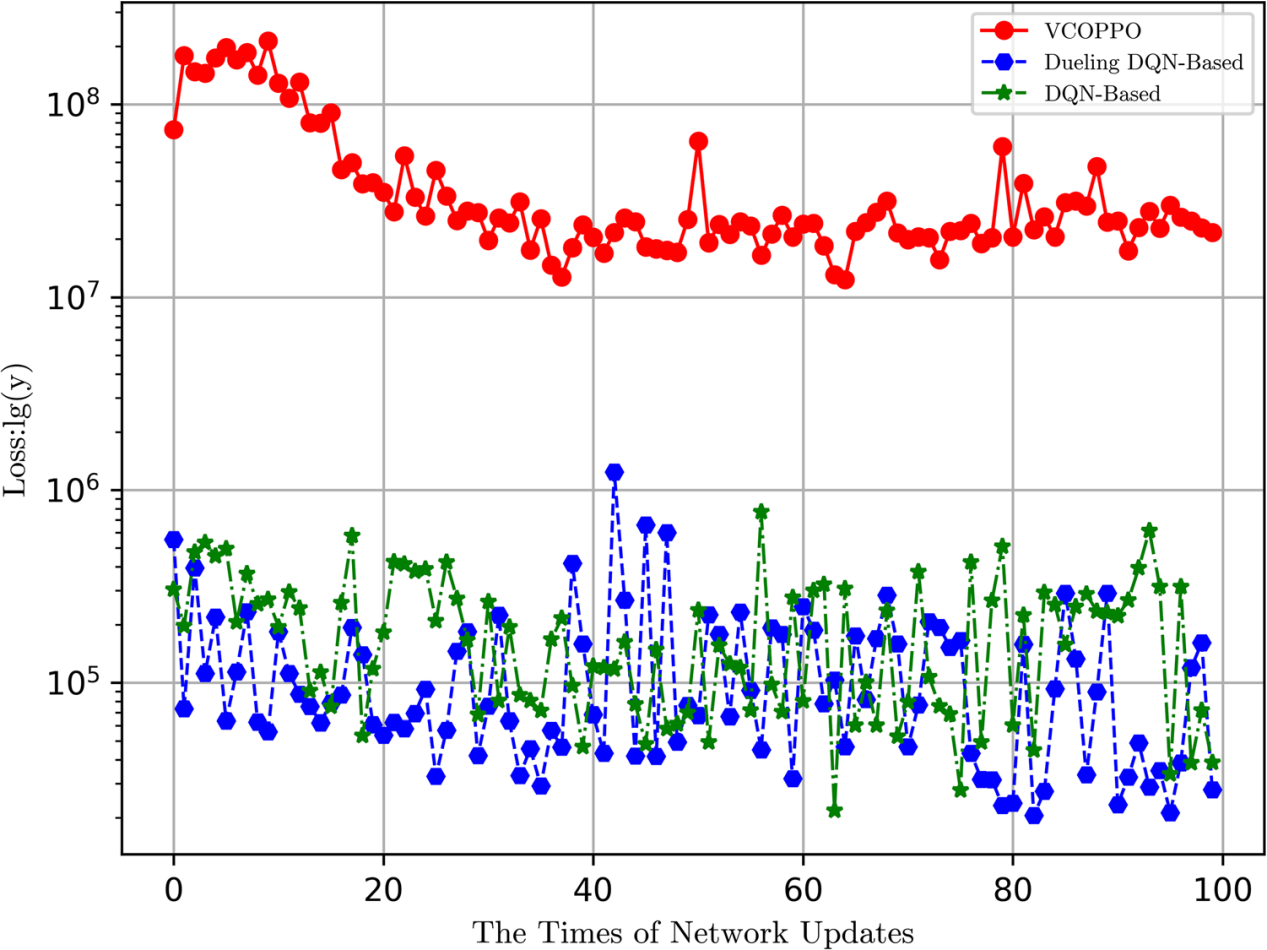
Comparison of loss functions with variety in times of update

Figure 5 further shows the convergence performance of the proposed VCOPPO, Dueling DQN, and DQN methods in logarithmic coordinates with the vertical coordinate at the base of 10. Here we only compare their convergence performance without comparing the value of loss because their loss functions are calculated in different ways, and we compare the losses under these three methods due to the fact that Q-learning and Random methods do not have the evaluation network. It can be seen that the loss of the proposed VCOPPO reduces as the number of network updates, and quickly reach convergence. On the contrary, the Dueling DQN, and DQN methods have a large float and can not reach convergence, so the target network and the evaluate network do not fit well, resulting in the above non-convergence on network reward and task processing delay. The results show that the proposed VCOPPO has better convergence performance in the scenario proposed in this paper.

**Fig. 6:**
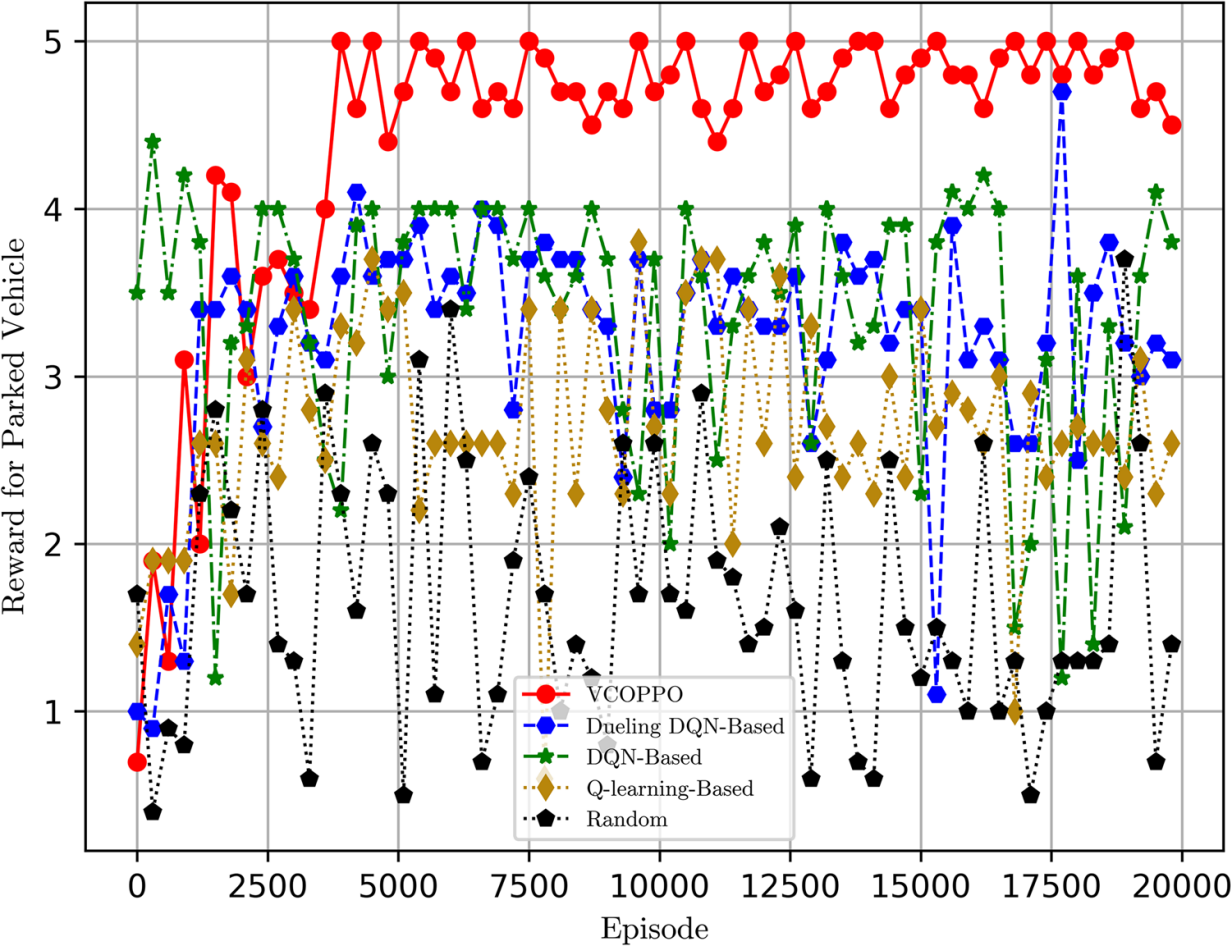
Changes in the unit price of the incentive

Figure 6 shows the changes in the unit price of the incentive, here the display is the sum of the unit price for completing ten tasks, It is observed that the unit price improves as the number of episodes increases until it attains a relatively stable value in the proposed VCOPPO, and the proposed VCOPPO converges to a large value compared to the other methods. In contrast, the Dueling DQN, DQN, Q-learning, and Random methods cannot reasonably adjust the unit price to the changing environment because the optimal action cannot be chosen in the decision. The results show that the proposed VCOPPO improves the incentive unit price by 37.5%, 29.4%, 63.1%, and 159.8%, compared to Dueling DQN, DQN, Q-learning, and Random methods, respectively. The higher the unit price, the more owners will be attracted to park their vehicles here to participate in the computation. That is, the proposed VCOPPO has more computational resources compared with other methods so it has advantages in network rewards and task processing delays, which proves the effectiveness of the proposed VCOPPO.

**Fig. 7:**
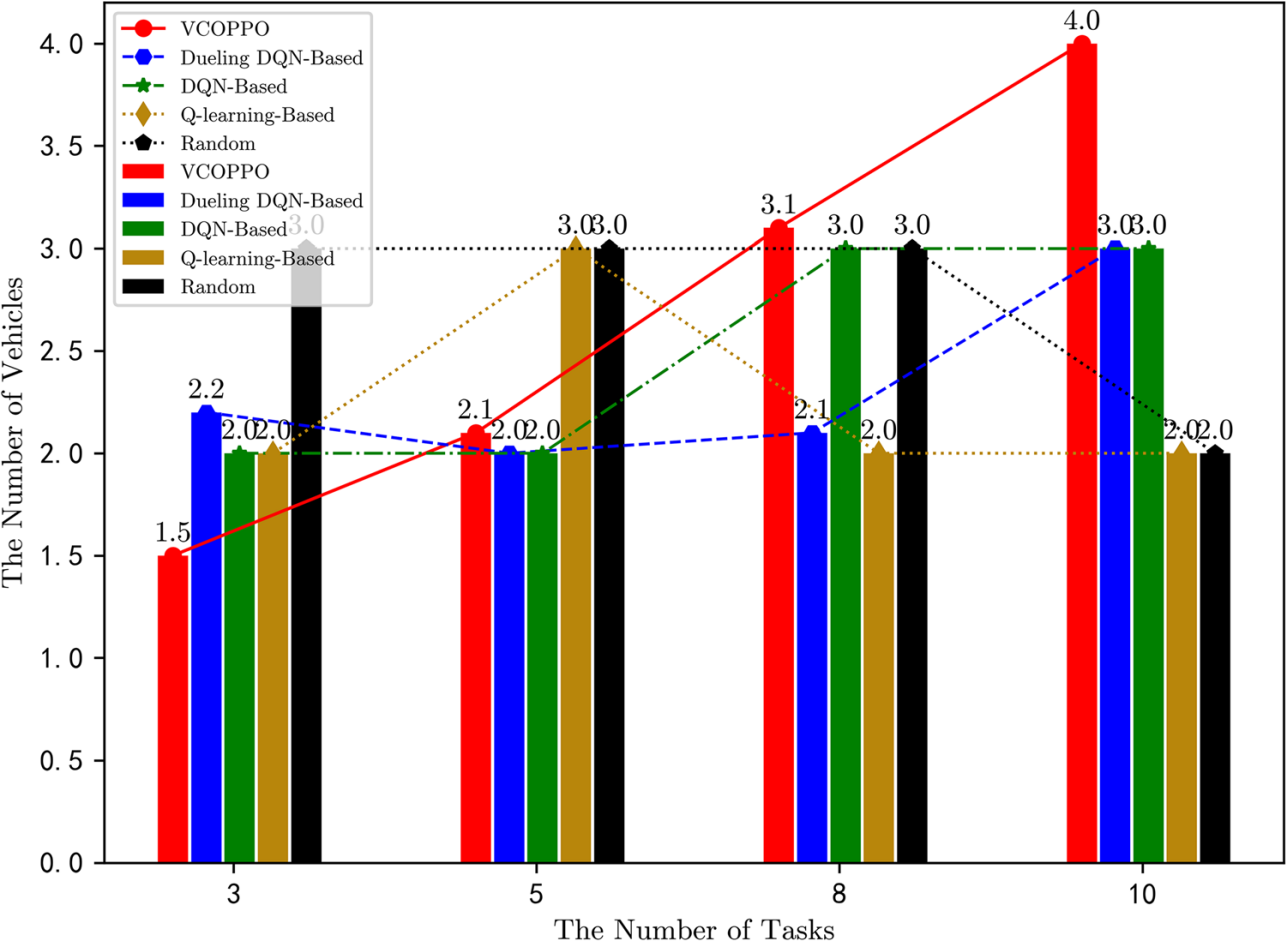
Comparison of the number of parked vehicles with variety the number of task

Figure 7 displays the variation of the number of vehicles involved in the task processing in the parking with the number of tasks, the number of vehicles here is the average of the number of vehicles that completed all tasks. In the proposed VCOPPO, it can be seen that fewer vehicles are involved in the task processing when the number of tasks is small, the number of vehicles involved in task processing increases as the number of tasks increases because the VEN requires more computational resources at this time. The number of vehicles under the Dueling DQN and DQN methods also increases with the number of tasks but does not reach a reasonable value when the number of tasks is large, this is because they choose the optimal action only in part of the decisions. However, the problem of the Q-learning method cannot select the correct action in the high-dimensional state-action space and the Random method selects actions randomly leading to them cannot properly adjust the number of vehicles according to the number of tasks. Thus, the proposed VCOPPO can effectively balance the output of VEN and the task processing delay by reasonably adjusting the number of parked vehicles when the number of tasks is variable.

## Conclusion

In this paper, we propose a vehicle-assisted computation offloading algorithm based on proximal policy optimization(VCOPPO) for User Equipment(UE) tasks using two types of vehicles as edge servers, combined with a dynamic reward mechanism and a computational resource allocation strategy. To obtain the optimal computation offloading decisions and resource allocation schemes, we formulate a non-convex optimization problem combining the Vehicle Edge Network(VEN) utility and the task processing delay and use the proposed VCOPPO to solve the non-convex optimization problem. Finally, we demonstrate through experiment that the proposed VCOPPO performs excellently in terms of network reward and task processing delay respectively, and can effectively schedule and allocate the computational resources of the parked vehicle. Compared with using Dueling Deep Q Network(Dueling DQN), Deep Q Network(DQN) and Q-learning methods, the proposed VCOPPO improves the network reward by 31%, 18% and 91%, reduces the delay in task processing by 78%, 63% and 74%, respectively. Therefore, it expands the computational resources near the UE and relieves the computational pressure on the network center.

The limitation of this paper is that we only consider the computation of a single road vehicle and the task can only be completely offloaded between the three computation ways. In future work, we will focus on the research that road vehicles can also jointly compute tasks and the tasks will be partially offloaded in each computation way.

## Data Availability

The manuscript does not contain any specific data set.
